# A Systematic Review of Waterborne Disease Outbreaks Associated with Small Non-Community Drinking Water Systems in Canada and the United States

**DOI:** 10.1371/journal.pone.0141646

**Published:** 2015-10-29

**Authors:** Wendy Pons, Ian Young, Jenifer Truong, Andria Jones-Bitton, Scott McEwen, Katarina Pintar, Andrew Papadopoulos

**Affiliations:** 1 Department of Population Medicine, University of Guelph, Guelph, Ontario, Canada; 2 FoodNet Canada, Centre for Food-borne, Environmental and Zoonotic Infectious Diseases, Public Health Agency of Canada, Ottawa, Ontario, Canada; 3 Laboratory for Foodborne Zoonoses, Public Health Agency of Canada, Guelph, Ontario, Canada; University of Coimbra, PORTUGAL

## Abstract

**Background:**

Reports of outbreaks in Canada and the United States (U.S.) indicate that approximately 50% of all waterborne diseases occur in small non-community drinking water systems (SDWSs). Summarizing these investigations to identify the factors and conditions contributing to outbreaks is needed in order to help prevent future outbreaks.

**Objectives:**

The objectives of this study were to: 1) identify published reports of waterborne disease outbreaks involving SDWSs in Canada and the U.S. since 1970; 2) summarize reported factors contributing to outbreaks, including water system characteristics and events surrounding the outbreaks; and 3) identify terminology used to describe SDWSs in outbreak reports.

**Methods:**

Three electronic databases and grey literature sources were searched for outbreak reports involving SDWSs throughout Canada and the U.S. from 1970 to 2014. Two reviewers independently screened and extracted data related to water system characteristics and outbreak events. The data were analyzed descriptively with ‘outbreak’ as the unit of analysis.

**Results:**

From a total of 1,995 citations, we identified 50 relevant articles reporting 293 unique outbreaks. Failure of an existing water treatment system (22.7%) and lack of water treatment (20.2%) were the leading causes of waterborne outbreaks in SDWSs. A seasonal trend was observed with 51% of outbreaks occurring in summer months (*p*<0.001). There was large variation in terminology used to describe SDWSs, and a large number of variables were not reported, including water source and whether water treatment was used (missing in 31% and 66% of reports, respectively).

**Conclusions:**

More consistent reporting and descriptions of SDWSs in future outbreak reports are needed to understand the epidemiology of these outbreaks and to inform the development of targeted interventions for SDWSs. Additional monitoring of water systems that are used on a seasonal or infrequent basis would be worthwhile to inform future protection efforts.

## Introduction

Small non-community drinking water systems (SDWSs) provide water to residents across Canada and the United States, as well as to transient populations of tourists and travellers at premises such as campgrounds, restaurants, and hotels [1]. These systems provide water to 15% of Canada’s population [2] and to 6% of the United States’ population [3].

The term “SDWS” can encompass a variety of water systems, including private and semi-private, public and semi-public, non-community, and micro systems. SDWS may refer to a water system servicing a small community or a single premises that is open to the public, such as a restaurant. A standard definition for SDWSs in North America does not exist. Current definitions used by legislative authorities differ, and are variably based on the number of people served by the water system, the number of connections to the system, the amount of water distributed (flow rate), the length of time the system is in use during the year, or the complexity of the system (this could range from a simple well and pump to a system consisting of coagulation, filtration and disinfection) [4]. For example, Health Canada defines systems that serve less than 500 individuals as “very small” and those serving 501 to 5000 individuals as “small” however, each province defines and regulates SDWSs differently from this categorization and from each other (S1 Table) [4–17]. In the United States (U.S.), a “very small” system serves 25 to 500 individuals and a “small” system serves 501 to 3,300 individuals [3]. Differences in definitions complicate the comparison of data from outbreaks that may have occurred in different jurisdictions. For the purpose of this review, a SDWS is defined as a non-community, privately or publicly owned system that provides drinking water to the visiting public.

Previous outbreak summaries have shown that in the United States [18] and Canada [19], roughly half of reported waterborne disease outbreaks are associated with SDWSs (44% and 62%, respectively). A report by the National Research Council found water systems in the United States serving fewer than 500 people exceeded microbial and chemical water standards twice as often as larger water systems [20]. The ability for small systems to address water safety risks is especially challenging. Larger water systems have comprehensive protection measures in place (e.g. multistep treatment systems with back-up measures in place or frequent monitoring practices), while small systems face the challenges of having relatively high operating costs associated with treatment for a small number of consumers [21], poor access to operator training [22–23], and low retention of knowledgeable water operators [22–23].

Since 1971, waterborne disease surveillance has been conducted regularly in the United States by the Centers for Disease Control and Prevention [24]. In Canada, there is no national surveillance system specific to waterborne illness and no standardized approach to data collection on sporadic or outbreak cases of waterborne illness, although there are enteric disease surveillance systems in place to inform our understanding of acute gastrointestinal infection [19]. Rates of waterborne disease, related to outbreaks and non-outbreaks, are significantly underreported because of the generally mild associated symptoms, short duration of illnesses, and lack of patient reporting to a physician, among other factors [25]. Majowicz et al. [26] estimated that over 300 cases of acute gastrointestinal illness (relating to both water and foodborne illnesses) occur in the community for every laboratory-confirmed case reported to a provincial laboratory in Canada.

Examination of past waterborne disease outbreaks is essential to understanding the causes of outbreaks, identifying system deficiencies, and informing future protection measures and risk mitigation strategies that are appropriate to the size of the water system [25]. While several recent reviews of waterborne disease outbreaks have been published in the United States and Canada [2], [18–19], [25], none focused on SDWSs using formal systematic review methods. The systematic review approach is a preferred approach to reviewing literature because it uses structured and transparent methods that aim to minimize bias, is reproducible, and provides reliable findings to inform decision-making [27], [28]; this can lead to more evidence-informed assessments of SDWS outbreaks.

This systematic review was undertaken to identify and analyze outbreak reports involving enteric pathogens occurring in SDWSs throughout Canada and the United States, from 1970 to 2014. The objectives of this study were to: 1) identify all published reports of waterborne disease outbreaks involving SDWSs in Canada and the United States since 1970; 2) identify the terminology used to describe SDWSs in these outbreak reports; and 3) summarize the reported factors contributing to these outbreaks, including water system characteristics, events surrounding the outbreaks, and the microorganism(s) involved.

## Methods

### Research questions and review protocol

This review was guided by the following research questions: “what were the causes of waterborne disease outbreaks in SDWSs in Canada and the United States from 1970–2014?” “what were the key characteristics of those outbreaks?” and “what terminology was used to describe SDWSs in these outbreak reports?”

A review protocol was created *a priori* outlining the search strategy, selection criteria, and screening and data extraction forms. The review was guided by a team of seven people with expertise in drinking water safety, public health, risk assessment, knowledge synthesis, and systematic review methodology.

### Search strategy

A comprehensive search algorithm was created. Firstly, key search terms were obtained from reviewing the titles and abstracts of 10 waterborne outbreak reports occurring in Canada and the United States in SDWSs [24], [29–37]. Terms were then combined in relevant categories (population, outcome and location) using Boolean logic operators. The search algorithm was tested in PubMed and a final algorithm was selected that retrieved the highest proportion of all known relevant articles.

The search algorithm was implemented in the following three bibliographic databases on June 20–21, 2013, with an updated search on July 17–18, 2014: PubMed/Medline, Web of Science, and Scopus. By way of example, the final algorithm as implemented in PubMed was as follows: ("gastroenteritis"[MeSH Terms] OR "gastroenteritis"[All Fields]) OR (“illness”[All Fields]) OR (“Outbreak”*) OR ("disease"[MeSH Terms] OR "disease"[All Fields]) AND (("waterborne") OR (("drinking water"[MeSH Terms]) AND "drinking water"))) AND (("Canada"[MeSH Terms] OR "Canada"[All Fields]) OR ("united states"[MeSH Terms] OR "united states"[All Fields]) OR (“North America” [MeSH Terms] OR “North America” [All Fields])); other database-specific algorithms are reported in the supplementary materials (S1 Fig). To minimize the risk of missing relevant outbreak reports, a search of Morbidity and Mortality Weekly Reports surveillance summaries and Canada Communicable Disease Reports was also conducted on July 31, 2013. The following grey literature sources were also searched using combinations of the key search terms: ProMED-mail and Google on October 30, 2013. These searches had no date restrictions and for pragmatic reasons the Google search was limited to the first 100 hits. All grey literature searches were updated on July 20, 2014. For search verification, the reference lists of five key summary and review articles [18–19], [25], [38–39] were also searched to identify further reports.

### Relevance screening

Screening of article titles and abstracts was conducted by two independent reviewers using a form that was developed *a priori* (S2 Table) and based on detailed inclusion/exclusion criteria (Table 1). The form was pre-tested by both reviewers on a selection of 20 abstracts before use in full screening. Screening proceeded when the kappa measure of agreement between reviewers was ≥0.8. Any reviewer disagreements were resolved by discussion until a consensus was achieved. The screening form was used to assess the relevance of titles and abstracts of all identified citations according to one key question: “Does the abstract investigate or discuss waterborne outbreak(s) in SDWSs in Canada or the United States occurring between 1970 and 2014?”.

**Table 1:** Inclusion and exclusion criteria used to identify relevant outbreaks occurring in small drinking water systems (SDWS) in this review.

Inclusion criteria	Exclusion criteria
Language: English, French, Spanish	Language: any language other than English, French, Spanish
Population: small non-community water systems, public or privately owned	Population: community or individual water supplies or if there was not enough information to determine the size of the water supply
	Study type: summary studies that do not provide detail on individual outbreaks
Outcome: 2 or more cases of acute gastrointestinal illness associated with a water supply	Outcome: single cases of illness associated with a water supply -contamination by non-enteric pathogens (e.g. Legionella), chemical contamination, and outbreaks associated with recreational water
Period: 1970- present	Period: pre- 1970

### Data extraction

Data extraction was conducted independently on full articles by two reviewers. The extraction form (S3 Table) was tested by both reviewers prior to use on five relevant articles and reviewer disagreements were resolved by discussion until a consensus was achieved. The form contained seven questions about the water system, including the type of water source (ground, surface, or mixed), whether the water system was operated seasonally (i.e. did not operate consecutively for 365 days/year), water treatment type, and terminology used to describe the water system’s size (including the term used to describe the water system size as an open text box, the number of connections to the water system, the flow rate and number of people served by the system). Outbreaks reported for a single premises (camp, restaurant, etc.) were attributed to a non-community system. Articles were excluded if insufficient information was provided to identify SDWS. Other data that were captured from the articles included: the date and location of the outbreak, the number of people ill, the reported/contributing events leading to the outbreak, the microbiological organisms involved, and the type of premises associated with the outbreak. Outbreak confirmation was also classified into categories defined by the Centres for Disease Control and Prevention (see Yoder *et al*. [24]): Class 1 (strength of evidence link- adequate epidemiologic and water quality data); Class 2 (strength of evidence link- adequate epidemiologic data, water quality data inadequate or not provided); and Class 3 (strength of evidence link—epidemiologic data provided but limited, laboratory confirmation of water quality data provided and adequate). A risk-of-bias assessment was not conducted due to inadequate reporting detail, as often occurs with outbreak reports [40].

### Systematic review management, data charting and analysis

Citations identified via the search were imported into the web-based reference management program RefWorks 2.0 (RefWorks, 2009), and duplicates were removed using the automatic de-duplication option. Citations were exported to an Excel spreadsheet for review (Microsoft Excel, 2003).

The data were summarized using descriptive tables and bubble charts. Bubble charts were used to graphically represent cross tabulations of data, where the size of the bubble was proportional to the number of outbreaks occurring in a given pair of variables [41]. Bubble charts were created for microorganism category involved in the outbreak by water source. These variables were chosen to explore relationships based on the findings of previous research [18], [25].

Data were descriptively analyzed in SPSS 20 (SPSS Inc., 2011) with unique outbreaks reported within articles as the unit of analysis. A chi-squared test was used to determine the statistical significance of differences in proportions of outbreaks between seasons (seasons were categorized as: winter- December, January and February; spring- March, April and May; summer- June, July and August; fall- September, October and November). Statistical significance was declared at *p ≤*0.05. Meta-analysis was not conducted as the primary aim of the study was to summarize outbreak characteristics. The Preferred Reporting Items for Systematic Reviews and Meta-Analyses (PRISMA) guidelines were followed in reporting the methods and results of this review (S4 Table) [42].

## Results

**Fig 1:**
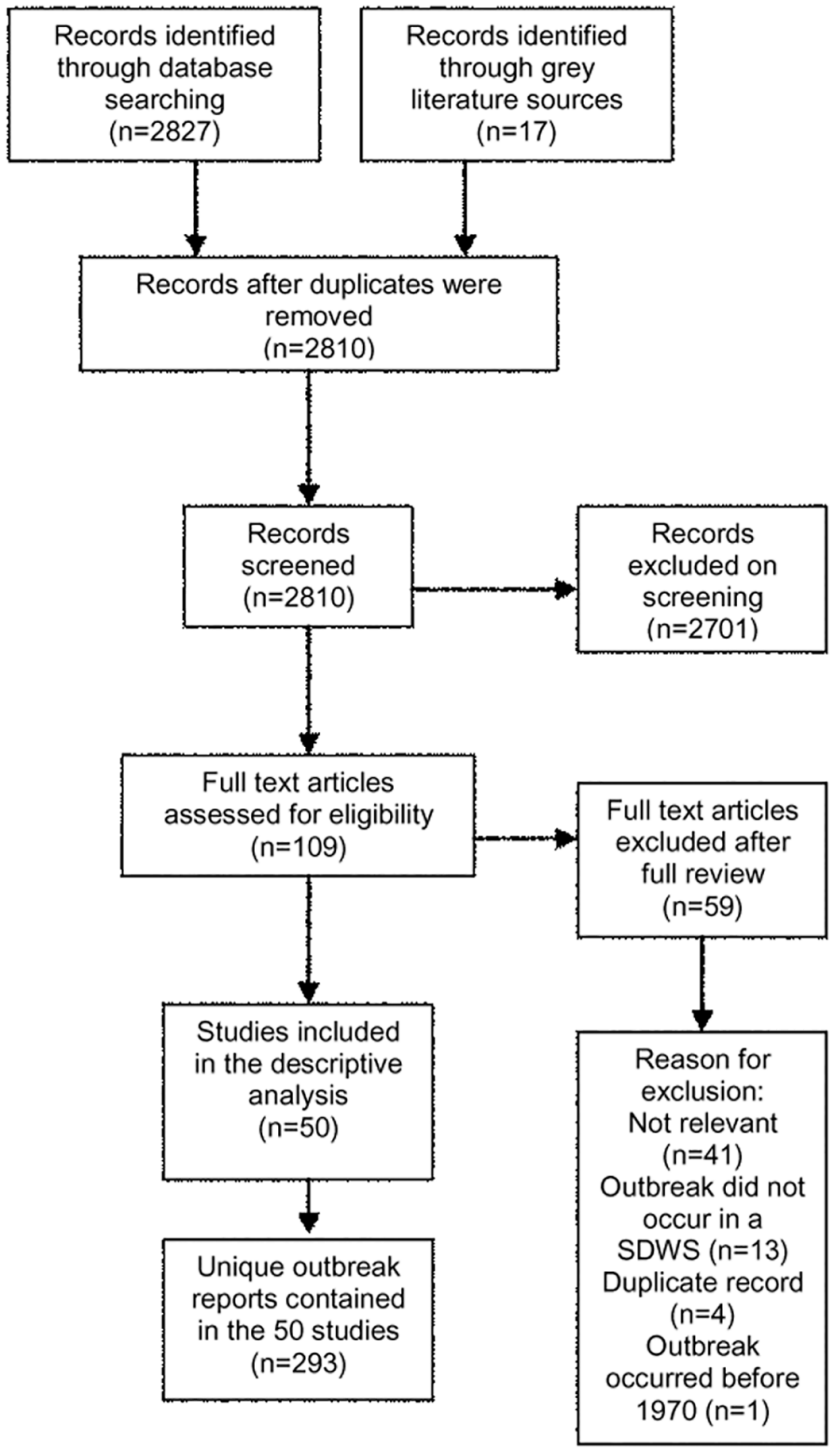
PRISMA flow chart detailing the screening of literature through the systematic review process. Reports of waterborne disease outbreaks in small drinking water systems in Canada and the United States between 1970–2014 were screened using this process.

The database and grey literature searches retrieved 2,810 unique citations that were screened for relevance. A total of 50 relevant articles were identified, which included 293 unique outbreak reports involving SDWSs (Fig 1). The descriptive summary and citation list of each outbreak is provided in S5 Table[43–71].

Of the 293 reported outbreaks, 121 (41.3%) occurred in Canada and 172 (58.7%) in the United States, from 1970 to 2014. The outbreaks resulted in a total of 41,862 illnesses and 3 deaths. The mean annual number of outbreaks reported over the 40-year period was 7.3, with a mean of 143 illnesses per outbreak. The number of illnesses per outbreak ranged from 2 to 5,000. In Canada, 3,992 illnesses were reported. When this number is scaled by the Canadian population it represents illness in 0.01% of the total population and 0.07% of the population served by small drinking water systems (3,992/35.5 million and 3,992/5.33 million, respectively). In the United States, 37,870 illnesses were reported. When this number is scaled by the United States population it represents illness in 0.01% of the total population and 0.2% of the population served by small drinking water systems (3,7870/321.7 million and 3,7870/19.3 million, respectively).

**Fig 2:**
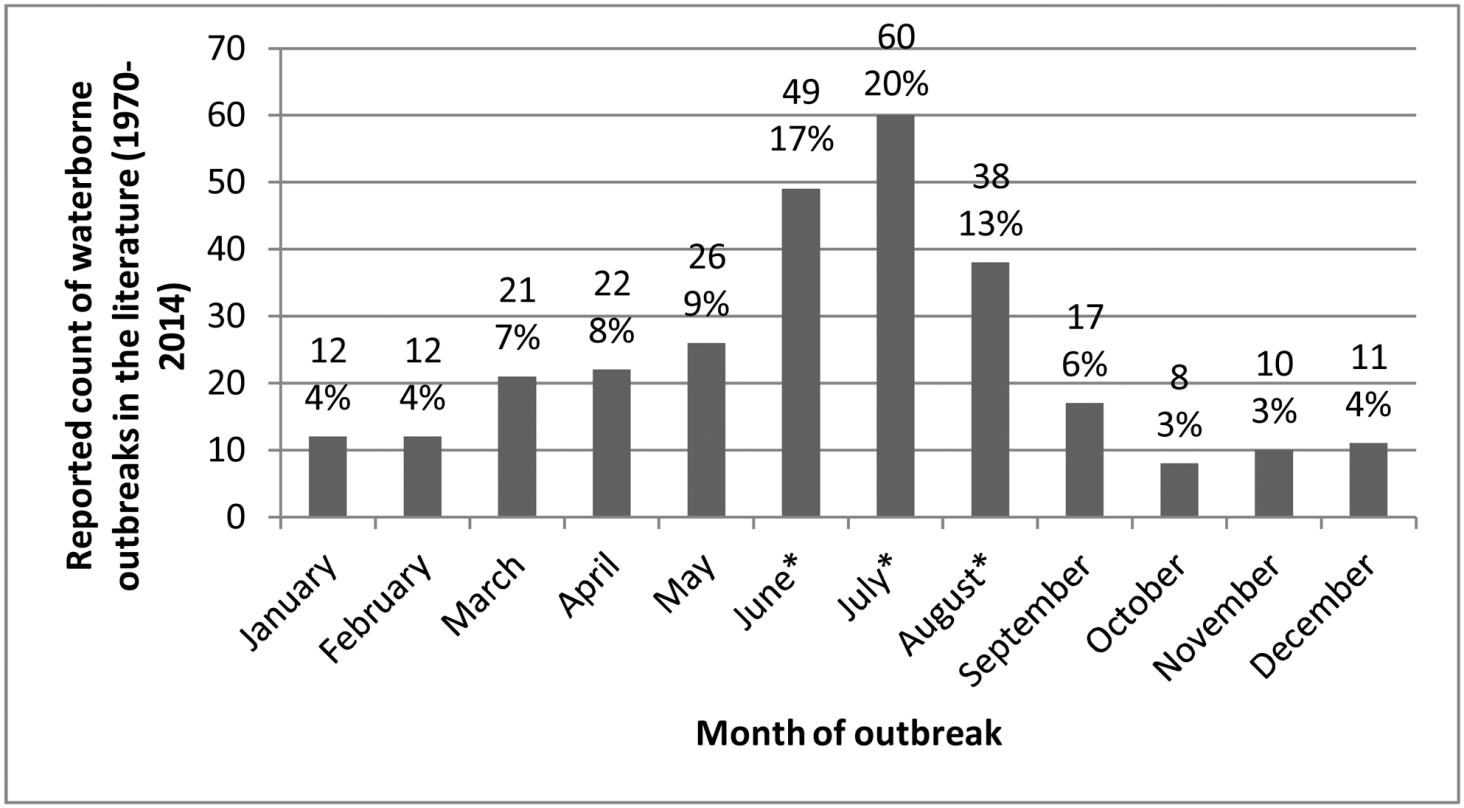
Frequency distribution of waterborne disease outbreaks in small drinking water systems (n = 286^). Outbreaks reported in the literature, by month, in Canada and the United States (1970–2014). *^Seven outbreak reports did not indicate the month during which the outbreak occurred*. *Count in summer months (June, *July*, *August) was significantly different (P < 0*.*001) from number of outbreaks occurring in other seasons*.

The numbers of outbreaks reported annually were greatest from 1985 to 1995 (187/293; 63.8%), with the highest number of outbreaks (31/293; 10.6%) reported in both 1991 and 1992. Outbreaks were reported throughout the year; however, a seasonal trend was observed with 51.4% (147/286) of outbreaks reported during the summer months (Chi-square = 112.01; *p*<0.001) (Fig 2).

A minority of reports identified how the outbreak was confirmed (99/293; 33.8%). Of these, 49.5% (49/99) were reported as having a Class 1 strength of evidence link, 6% (6/99) as having a Class 2 strength of evidence link, 32.3% (32/99) as having a Class 3 strength of evidence link; 11.1% (11/99) utilizing both stool and water samples, and 1% (1/99) water samples only.

**Fig 3:**
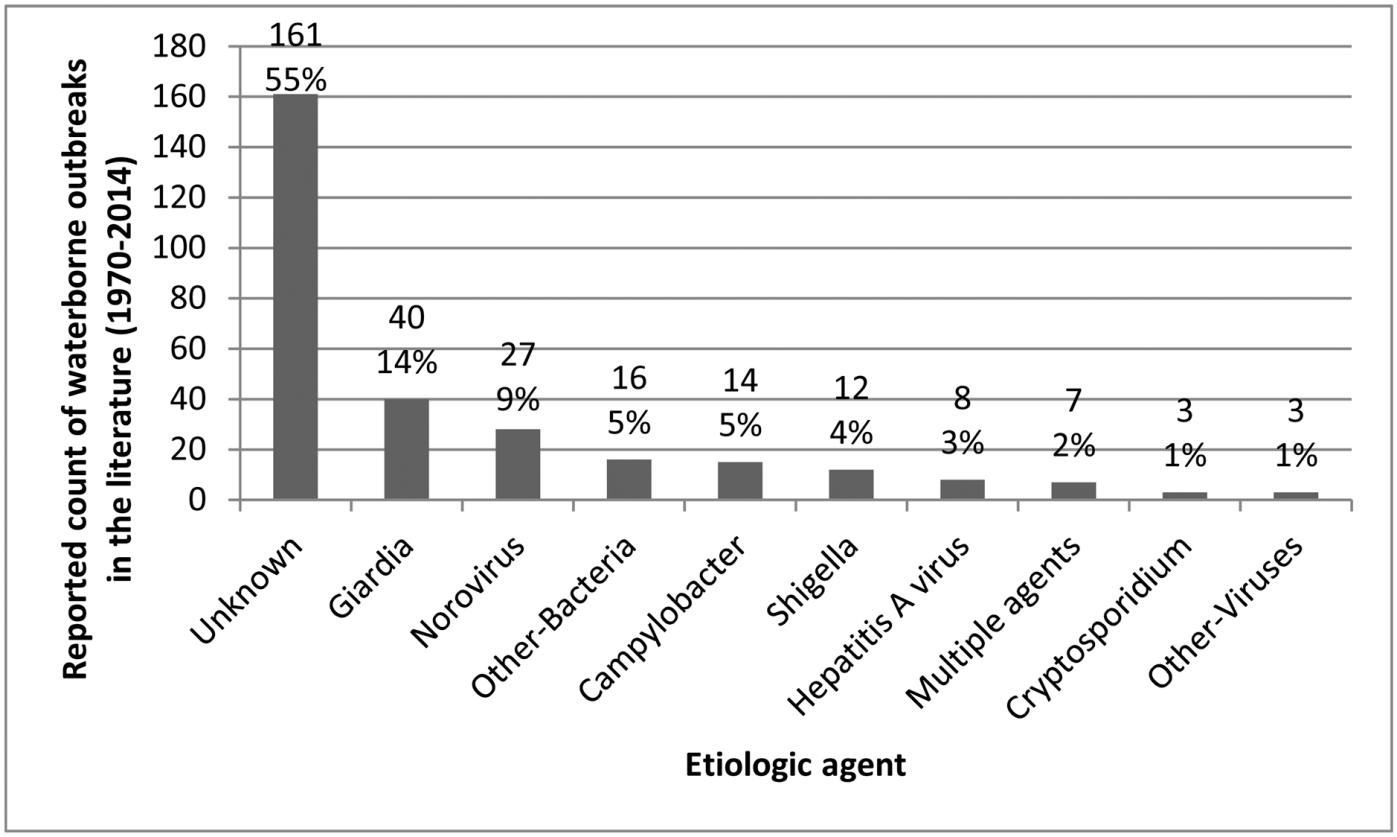
Frequency distribution of etiologic agents in waterborne disease outbreaks in small drinking water systems. Outbreaks reported in Canada and the United States (1970–2014) (n = 293). *Other bacteria include*: *Salmonella spp* (n = 6), *E*.*coli O157*:*H7* (n = 3), *Yersinia enterocolitica* (n = 3), *Streptococcus spp* (n = 2), *Bacillus cereus* (n = 1) *and Pseudomonas aeruginosa* (n = 1). *Other viruses include*: *Small Round Structured Viruses (SRSV)* (n = 1) *and Rotavirus* (n = 2). *Multiple agents included*: *Campylobacter jejuni* and *E*. *coli O157*:*H7* (n = 2); *Campylobacter jejuni* and *Giardia intestinalis* (n = 2); *Campylobacter jejuni* and *Shigella sonnei* (n = 1); *Campylobacter jejuni* and Norovirus (n = 1); and *Campylobacter jejuni* and *Yersinia enterocolitica* (n = 1).

Slightly more than half of the outbreaks were of undetermined etiology (161/293; 54.9%). *Giardia intestinalis* was the most commonly identified agent (42/293; 14.3%), followed by norovirus (29/293; 9.9%) and *Campylobacter jejuni* (22/293; 7.5%) (Fig 3). Seven outbreaks (2.4%) involved multiple agents (Multiple agents included *Campylobacter jejuni* and *E*. *coli O157*:*H7* (n = 2); *C*. *jejuni* and *G*. *intestinalis* (n = 2); *C*. *jejuni* and *Shigella sonnei* (n = 1); *C*. *jejuni* and Norovirus (n = 1); and *C*. *jejuni* and *Yersinia enterocolitica* (n = 1)).

**Fig 4:**
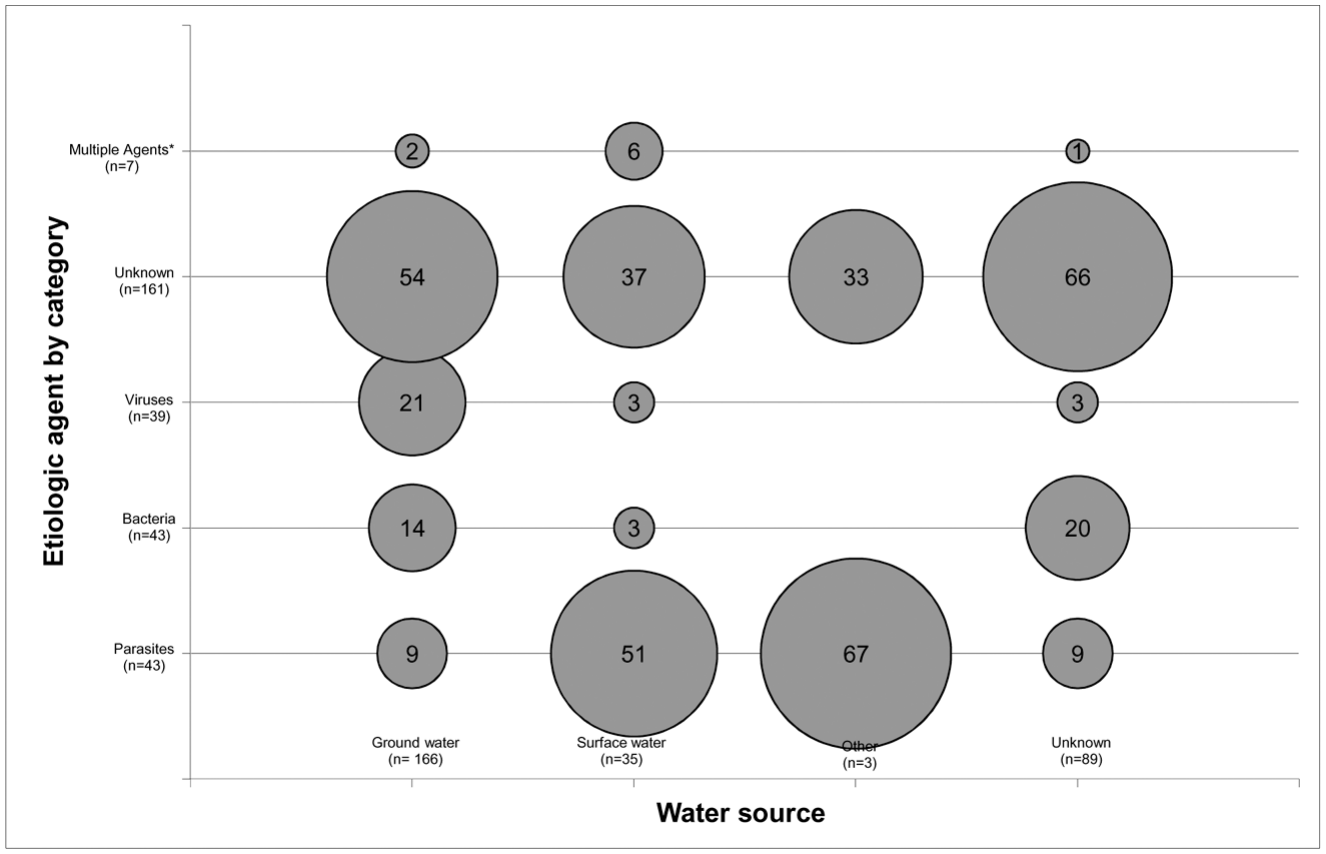
Bubble chart of reported waterborne disease outbreaks (n = 293) in small drinking water systems. Outbreaks in Canada and the United States (1970–2014) stratified by water source and category of causative microorganism. Bubbles show the column percentages for each water source category, and their sizes are proportional to these values. *Other water sources include cistern and reservoir.

*Giardia intestinalis* and Norovirus were determined to have caused the two largest proportions of human cases of illness (26.9% and 24.2%, respectively) (Table 2). In ground water sources (Fig 4), parasites, bacteria, and viruses were associated with 19.2% (15/78), 30.8% (24/78) and 44.9% (35/78), respectively, of outbreaks where an etiological agent was identified. In surface water sources, parasites were associated with 81.8% (18/22) of outbreaks of known etiology, while bacteria and viruses were each implicated in 4.5% (1/22).

**Table 2:** Summary of number of illnesses and deaths by causative microorganism as reported in 293 waterborne disease outbreaks involving small drinking water systems in Canada and the United States (1970–2014).

Microorganism	Count of ill^1^ # (%)	Deaths
*Giardia intestinalis*	11 931(26.9)	0
Norovirus	10 739(24.2)	2
*E*. *coli O157*:*H7*	3 050(7.3)	0
Campylobacter	3 069(6.9)	0
*Shigella sonnei*	1 863(4.5)	0
*Cryptosporidium parvum*	650(1.6)	0
Other^2^	590(1.4)	0
Hepatitis A virus	273(0.7)	0
Unknown^3^	12 210(29.3)	1
Total^4^	44 375	3

^1^Attack rate was not included as it was not available for the majority of outbreak reports

^2^Other microorganisms included *Salmonella spp*., Small Round Structured Viruses (SRSV), Rotavirus, *Yersinia enterocolitica*, *Streptococcus spp*., *Bacillus cereus and Pseudomonas aeruginosa*

^3^Unknown was not reported

^4^Count exceeds the total number of people ill due to illness associated with multiple agents

A total of 30.3% (89/293) of reports did not indicate the type of water source that was involved in the outbreak. Of the outbreak reports that indicated the implicated water source, 81.4% (166/204) identified ground water (including wells and springs), 18.1% (37/204) identified surface water (including lakes, ponds, rivers, creeks, streams, and reservoirs); the remaining outbreak occurred in a cistern (1/204).

**Fig 5:**
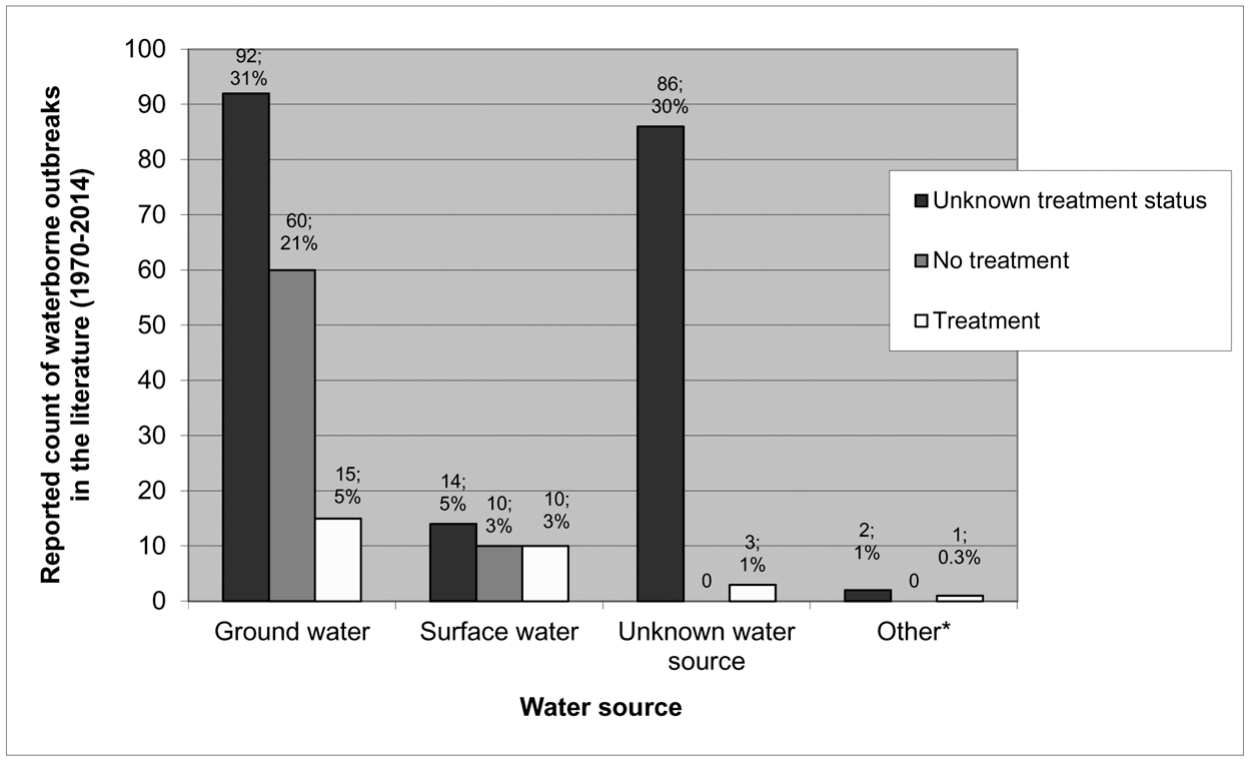
Frequency distribution of reported waterborne disease outbreaks (n = 293) in small drinking water systems. Outbreaks in Canada and the United States (1970–2014) stratified by treatment and water source. *Other water sources include cistern and reservoir.

Approximately 66% (194/293) of outbreak reports did not indicate whether there was water treatment in place at the time of the outbreak. When this information was reported, 70.7% (70/99) of implicated water supplies did not have treatment in place (Fig 5). The most common type of reported treatment was chlorination only (79.3%; 17/29), followed by filtration and chlorination (20.7%; 6/29). Another 20.7% (6/29) of reports indicated treatment was used but did not specify the method employed.

Factors contributing to outbreaks, where reported, were categorized into seven types (Table 3). The most common deficiencies reported (of the 170/293 reports where a deficiency was reported) included inadequate treatment (74/170; 43.5%) and lack of water treatment (66/170; 38.8%). Multiple deficiencies were reported in 15.9% (27/170) of outbreak reports.

**Table 3:** Water system failure (n = 326) cited in 293 waterborne disease outbreak reports in small drinking water systems in Canada and the United States (1971–2014), stratified by water source.

Deficiency^1^	Description	Water Source				
		Ground Water# (%)	Surface Water# (%)	Unknown# (%)	Other^2^# (%)	Total
Inadequate treatment	An existing water treatment system failed to provide adequate protection from contamination	55 (28.8)	13 (31.7)	3 (3.3)	3 (75.0)	74
Lack of treatment	No water treatment was used	57 (29.8)	9 (22.0)	0	0	66
Sewage contamination	Human sewage contaminated the water supply at any point in the water system	21 (11.0)	4 (9.8)	3 (3.3)	0	28
Distribution system	Contamination entered through the distribution system e.g. cross connections, broken water main	14 (7.3)	1 (2.4)	2 (2.2)	0	17
Source contamination	Contamination from surrounding land use, principally animal waste	2 (1.0)	3 (7.3)	0	1 (25.0)	6
Weather events	Extreme weather events, such as heavy precipitation or prolonged drought, contributed to contamination	2 (1.0)	1 (2.4)	0	0	3
Unknown^3^		31 (16.2)	10 (24.4)	82 (91.1)	0	123
Other^4^		9 (4.7)	0	0	0	9
Total		191	41	90	4	326

^1^27 outbreak reports noted more than one deficiency

^2^Other water sources included a cistern (n = 1) and reservoirs (n = 2)

^3^Unknown was not reported

^4^Other deficiencies included contaminated water storage, point of use contamination, geological conditions (i.e. soil conditions that led to the contamination of ground water), well construction and contaminated filter sand

**Fig 6:**
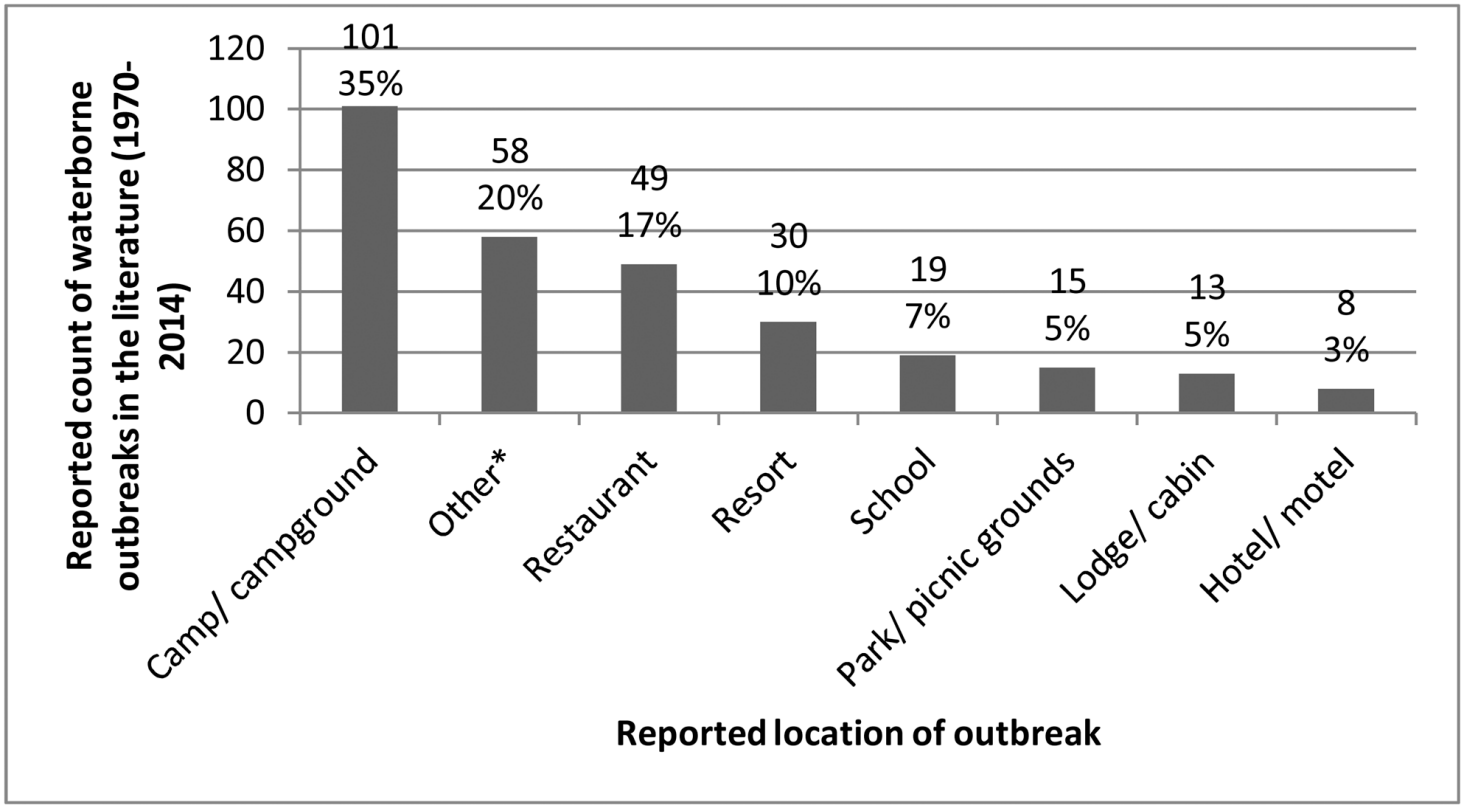
Premises type of reported waterborne disease outbreaks (n = 293) in small drinking water systems. Outbreaks in Canada and the United States (1970–2014). *Other premises included tourist attraction (n = 11), special event (n = 10), retirement home (n = 7), recreation centre (n = 4), church (n = 4), cafeteria/catering (n = 4), ranch (n = 3), workplace (n = 3), hospital/daycare (n = 3), military base (n = 3), country club (n = 2), trailer park (n = 2), prison (n = 1), and supermarket (n = 1).

Few outbreak reports provided detailed information on the implicated water system, including the number of people served (described in 9/293 reports; 3.1%), the number of connections in the system (5/293; 1.7%), and the water system’s flow rate (3/293; 1%). No outbreak reports indicated whether the water system was seasonal. All outbreak reports included the premises type (Fig 6).

The term “non-community water system” was most commonly used when describing the type of SDWS (135/293; 46%); 2.4% (7/293) described the system by the premises it served, for instance “the water supply for the resort”, and 1.7% (5/293) each used the terms “private water supply” and “individual water system”. Almost half of reports (141/293; 48.1%) did not classify the type of SDWS that was involved in the outbreak.

## Discussion

The identification of outbreaks involving small systems is challenging given the typically small number of people exposed and the transient population they often serve, who are less likely to report illness [72]. This study used systematic and transparent review methods to summarize the characteristics, other available information and contributing factors associated with reported waterborne disease outbreaks involving SDWSs in Canada and the United States from 1970 to 2014. Many of the identified factors have previously been identified to cause outbreaks in any drinking water system, regardless of size. Previous studies in the United States and Canada [73–75] have found untreated and inadequately treated ground water to be the most common factors leading to outbreaks in small drinking water supplies. In this study, outbreaks due to suspected or confirmed infectious agents in SDWSs were most commonly attributed to the failure of an existing water treatment system (22.7%), followed by the absence of water treatment (20.2%).

Small drinking water supplies commonly utilize ground water sources [76]. While it is generally believed that ground water is better protected from contamination than surface water [75], ground water quality is heavily influenced by several factors including the type of well (dug vs. drilled), proper construction and placement of wells, infiltration into shallow or deteriorating wells (ground water under the direct influence of surface water), and contamination from agriculture, septic or wildlife sources [75]. The need for appropriate drinking water treatment, regardless of the water source, is highlighted in this review. The adoption of drinking water treatment by all SDWSs is a critical step to ensure safe water supplies for these communities, and would serve to reduce the incidence of waterborne outbreaks [77–78]. Season continues to be identified as a factor in the incidence of waterborne outbreaks [18]. A larger proportion of the outbreaks in this review (51%) occurred in the summer months compared to other seasons. A review of ground water pathogen control studies in Canada and the United States [79] found an association between viral presence and precipitation patterns. This review found that viruses were associated with 45% of outbreaks in ground water supplies. Seasonality in outbreak occurrence may be the result of increased pathogen prevalence in source waters, coupled with contributing weather events such as heavy rains or drought [80], but it may also reflect the seasonal use of some SDWSs. Half of the outbreaks in this review were located in camps/campgrounds/cabins/parks. It is likely that many of these systems operate on a seasonal basis however, none of the publications reviewed reported if the water system was used in a seasonal manner. Water systems that are used on a seasonal or infrequent basis, such as summer camps, trailer parks and campgrounds, face high demands on the water supply from many visitors during short periods of time [81]. Supplies that provide water on a seasonal basis require the same level of ongoing monitoring and response to adverse test results as year-round systems, when they are in use. Additional monitoring of these systems would be worthwhile in order to further investigate water quality and treatment in small water systems. Retrospective evaluation of SDWS outbreak reports can improve our knowledge about key deficiencies common across outbreaks, while identifying important knowledge and reporting gaps, to inform future prevention efforts. In order for this to be successful however, the reporting framework must be detailed enough to ensure the information available in the peer-reviewed and grey literature can be meaningfully interpreted and analysed [82]. A large number of reports included in this review lacked detailed information on the SDWS, including detail on the cause of the outbreak (37.7%) and the etiological agent involved (55%). Waterborne outbreaks can be difficult to recognize and investigate; often outbreak investigations are time-sensitive and delays in the onset of symptoms or in getting started can hinder the environmental investigation into the probable source and cause of illness [19], [25]. The identification of etiologic agents in both exposure sources (water) and human stools has improved over time with enhanced laboratory diagnostic tools, specifically with the more readily available capability to detect Norovirus [18], [83]. However, Craun et al. found in the United States, that the number of outbreaks reporting the cause of the outbreak as ‘unknown’ has increased over time [18]. This observation may be influenced by a lack of a systematic mechanism for reporting details associated with a waterborne outbreak. Also, investigations are hindered by the environmental conditions and the transient nature of water contamination, sometimes making identification of the cause of the outbreak extremely difficult or impossible. The high frequency of missing information from outbreak investigations identified in our research highlights the need for timely, comprehensive investigation of waterborne disease outbreaks and improved outbreak reporting practices that include explanations for missing information (e.g. whether missing pathogen data was due to negative test results, or limited or no testing).

We also found great variation in the terminology used to describe the size of the water system involved in SDWS outbreaks. The term “non-community” was commonly used to describe the water system involved in outbreaks occurring in the United States. This term is used in the United States to denote systems that serve the general public and have 15 or more connections, or serve an average of 25 or more people [84]. Slightly over half of the reports (54.4%) described the characteristics and ownership of the system in the report, or referred to the water system as a “private” or “individual” system. Individual water systems that serve a single private residence are also commonly referred to as private water systems; this can be misleading and reduces the usefulness of outbreak summaries using this terminology. A standard definition of SDWSs across jurisdictions would facilitate the retrospective analysis of waterborne disease outbreak data to identify common themes and risk factors, to inform future interventions. Since a common definition of SDWSs does not exist within Canada or the United States, it would be helpful if outbreak reports would include information on water system characteristics to facilitate comparisons between and within jurisdictions. In the United States, there are national reporting guidelines for waterborne disease outbreaks [85]; however, many characteristics of the water systems are not collected (e.g. seasonality, number of connections and flow rate). The collection of information on drinking water outbreaks in Canada is not standardized [19]. The development of standardized reporting guidelines for waterborne outbreaks, regardless of the system size, would help inform the evaluation of public health risk in these systems and intervention efficacy. Specific recommendations for enhancing outbreak reporting in SDWSs are outlined in Table 4.

**Table 4:** Recommended items to include when reporting outbreaks associated with small drinking water systems.

Category	Recommendation
Cause of the outbreak	Number of probable and confirmed cases
	Microorganism(s) involved in the outbreak (identify as suspected or confirmed)
	Contributing cause(s) of the outbreak
	Investigation methods used to confirm outbreak (e.g. stool or water samples, interviews of ill persons, or water system assessment)
	Environmental sampling information (water testing, methods used, number of samples collected, time delay between outbreak notification and sample collection, local hydrogeology)
Size of the water system	Number of people served per day/week
	Flow rate
	Number of connections to the water supply
Characteristics of the water system	Water source (e.g. ground or surface) and description (e.g. well or spring)
	Water treatment (type of disinfection and filtration specified)
	Setting of exposure (i.e. premises type)
	Months the water system is in operation

### Study Limitations

Reporting and publication bias may have resulted in under-reporting of small outbreaks in the literature, given that large outbreaks are more likely to be investigated and published. Articles that did not explicitly mention the terms “outbreak” or “drinking water” could have been missed in the database searches, and since the terminology used to describe SDWSs varied widely across reports, it is possible that outbreak reports could have been missed. However, the verification strategy of reviewing the reference lists of other outbreak reviews aimed to minimize the number of potentially missed outbreak reports. Misclassification bias may have also occurred; based on our approach to identifying SDWSs in reports, some outbreaks could have been overlooked or outbreaks that were not actually associated with a SDWS could have been included during data extraction. However the detailed inclusion/ exclusion criteria used in this study attempted to minimize this. Analysis of data was limited by the large amount of missing data in many of the outbreak reports.

## Conclusion

This review used a systematic and transparent approach to identify and characterize published waterborne disease outbreak reports involving SDWSs in Canada and the United States from 1970 to 2014. We found great variability in the terminology used to describe a SDWS and a lack of reporting of important details about the water systems and causes of outbreaks in these reports. More consistent reporting and descriptions of SDWSs in future outbreak reports would help to identify common risks, which would help to inform targeted public health interventions. This review found that lack of water treatment and inadequate treatment was a leading cause of outbreaks involving SDWSs. Outbreaks were commonly associated with water systems that tend to be used on a seasonal or infrequent basis, such as summer camps, trailer parks and campgrounds. Additional monitoring of these types of systems would be worthwhile to inform future protection efforts.

## Supporting Information

S1 Fig Search strategy used to identify reported drinking water outbreaks in small drinking water systems in Canada and the United States (1970–2014). (DOC)

S1 Table Definitions of small drinking water systems across Canada. (DOCX)

S2 Table Relevance screening form used in identifying reported waterborne disease outbreaks in small drinking water systems in Canada and the United States (1970–2014). (DOC)

S3 Table Form used to characterize and extract the data from relevant articles reporting waterborne disease outbreaks in small drinking water systems in Canada and the United States (1970–2014). (DOC)

S4 Table Article adherence with Preferred Reporting Items for Systematic Reviews and Meta-Analyses guidelines for reporting systematic reviews. (DOC)

S5 Table Descriptive summary of waterborne disease outbreaks included in the systematic review. (XLS)

## Abbreviations

SDWS: Small non-community drinking water system
U.S.: United States
