# Supplementary material for: A Systematic Review of Waterborne Disease Outbreaks Associated with Small Non-Community Drinking Water Systems in Canada and the United States
Source: PLoS One. 2015 Oct 29;10(10):e0141646. doi: 10.1371/journal.pone.0141646 (PMC4625960; doi:10.1371/journal.pone.0141646)
Supplement: S1 Fig — (DOC) [file pone.0141646.s001.doc]

**S1 Fig. Search strategy used to identify reported outbreaks in small drinking water systems in Canada and the United States (1970-2014).**

**PubMed**

Date of search: June 19, 2013

Hits: 536

| **Search Terms** |
| --- |
| (((("gastroenteritis"[MeSH Terms] OR "gastroenteritis"[All Fields]) OR (illness[All Fields]) OR (Outbreak*) OR ("disease"[MeSH Terms] OR "disease"[All Fields]))) AND (("waterborne") OR (("drinking water"[MeSH Terms]) AND "drinking water"))) AND (("canada"[MeSH Terms] OR "canada"[All Fields]) OR ("united states"[MeSH Terms] OR "united states"[All Fields])) |
| Restrictions: 1970-2013 |
|  |

Date of search: July 17, 2014

Hits: 124

| **Search Terms** |
| --- |
| (((("gastroenteritis"[MeSH Terms] OR "gastroenteritis"[All Fields]) OR (illness[All Fields]) OR (Outbreak*) OR ("disease"[MeSH Terms] OR "disease"[All Fields]))) AND (("waterborne") OR (("drinking water"[MeSH Terms]) AND "drinking water"))) AND (("canada"[MeSH Terms] OR "canada"[All Fields]) OR ("united states"[MeSH Terms] OR "united states"[All Fields] OR (“North America”[MeSH Terms] or “North America”[All Fields]))) |
| Restrictions: 2013-2014 |

**Web of Science**

Date of search: June 20, 2013

Hits: 397

| **Search Terms** |
| --- |
| (TS=(disease* OR illness* OR gastroenteritis OR outbreak*) OR TI=(disease* OR illness* OR gastroenteritis OR outbreak*)) AND (TS=("drinking water" OR waterborne) OR TI=("drinking water" OR waterborne)) AND (TS=(Canada OR US) OR TI=(Canada OR US)) |
| Restrictions= 1970-2013 |
| TS=topic  TI=title |

Date of search: July 17, 2014

Hits: 33

| **Search Terms** |
| --- |
| TI=(disease* OR illness* OR gastroenteritis OR outbreak*)) AND TI=("drinking water" OR waterborne)) AND (TI=(Canada OR US OR “North America”)) |
| No restrictions |
| TI=title |

**Scopus**

Date of search: June 20, 2013

Hits: 1287

| **Search Terms** |
| --- |
| (disease* OR illness* OR gastroenteritis OR outbreak*) AND (("drinking water" OR waterborne)) AND (Canada OR "united states") |
| No restrictions |
| Search title-abstract-keyword |

Date of search: July 18, 2014

Hits: 74

| **Search Terms** |
| --- |
| (disease* OR illness* OR gastroenteritis OR outbreak*) AND (("drinking water" OR waterborne)) AND (“North America”) |
| Restrictions 2013-2014 |
| Search title-abstract-keyword |

**ProMED-mail**

Date of search: June 20, 2013

Hits: 6 and 0

| **Search Terms** |
| --- |
| waterborne AND USA  waterborne AND Canada |
| No restrictions |
|  |

Date of search: July 20, 2014

Hits: 11 and 0

| **Search Terms** |
| --- |
| waterborne AND USA  waterborne AND Canada |
| No restrictions |
|  |

**Canada Communicable Disease Registry**

Date of search: July 31, 2013

Hits: 0

| **Search Terms** |
| --- |
| No search tool available, instead a review of all article titles was conducted |

Date of search: July 20, 2014

Hits: 0

| **Search Terms** |
| --- |
| No search tool available, instead a review of all article titles from 2013-2014 was conducted |

**Morbidity and Mortality Weekly Report**

Date of search: July 31, 2013

Hits: 188

| **Search Terms** |
| --- |
| “Drinking water” AND Outbreak |

Date of search: July 20, 2014

Hits: 188

| **Search Terms** |
| --- |
| “Drinking water” AND Outbreak |

**Google**

Date of search: October 30, 2013

Hits: 1,470,000 and 1,760,000

| **Search Terms** |
| --- |
| 1. “Drinking water” AND Outbreak AND Canada 2. “Drinking water” AND Outbreak AND “United States” |

Date of search: July 20, 2014

Hits: 1,130,000 and 1,960,000

| **Search Terms** |
| --- |
| 1. “Drinking water” AND Outbreak AND Canada 2. “Drinking water” AND Outbreak AND “United States” |
