# Supplementary material for: A Systematic Review of Waterborne Disease Outbreaks Associated with Small Non-Community Drinking Water Systems in Canada and the United States
Source: PLoS One. 2015 Oct 29;10(10):e0141646. doi: 10.1371/journal.pone.0141646 (PMC4625960; doi:10.1371/journal.pone.0141646)
Supplement: S1 Table — (DOCX) [file pone.0141646.s002.docx]

**S1 Table. Definitions of** **small drinking water systems** **across Canada.**

| **Province** | **Terminology** | **Definition** |
| --- | --- | --- |
| Alberta [5] | Small water system | Serves a municipal or privately owned public facility, with a population of less than 500.  Surface water or ground water under the influence of surface water is excluded from the small systems category. |
| British Columbia [6] | Small water system | Water supply system that serves up to 500 individuals during any 24-hour period. |
| Manitoba [7] | Semi-public water systems | Systems with less than 15 service connections or a public facility such as a school or hospital with its own water supply. |
| New Brunswick [8] | Water works  (Does not specifically identify small systems) | All or any part of a private, public, commercial or industrial works for the collection, production, treatment, storage, supply or distribution of water.  Includes all municipal drinking water systems, those owned by the crown, and those privately owned |
| Newfoundland and Labrador [9] | Very small system  Small system | Serve populations of 500 or fewer people.  501-1500 people |
| Northwest Territories [10] | **Small system** | Water treatment plants are classified according to the complexity of the treatment system, the quality of the raw water source, capacity of the system, types and degrees of process controls and instrumentation, chemicals used in the treatment process and the treatment and disposal of waste such as backwash water, sludge and residuals. Small systems are the simplest systems in the NWT. |
| Nova Scotia [11] | Public drinking water supply  (Does not specifically identify small systems) | A water works system for the provision of piped water for human consumption where the system has at least 15 service connections or serves 25 or more individuals per day at least 60 days of the year. This includes municipal, commercial, institutional, industrial, and privately owned water supplies |
| Nunavut [12] | Unknown | There is no requirement for individual systems to provide public reporting |
| Ontario [13] | Small drinking water system | A business or premise that makes drinking water available to the public and does not get their drinking water from a municipal drinking water system. |
| Prince Edward Island [14] | Small public drinking water facility | Serves 100 or fewer customers |
| Quebec [15] | Very small systems  Small systems | 21 to 200 people  201 to 1000 people |
| Saskatchewan [16] | Semi-private waterworks | A flow of less than 18,000 litres per day. Such as on-site water systems serving restaurants, motels, campgrounds, small parks, municipal wells with no distribution system. |
| Yukon [17] | Small drinking water system | A system other than a large drinking water system, that provides drinking water that:  (a) may have a water source or obtain drinking water from a large drinking water system; (b) has infrastructure that collects, produces, treats or stores drinking water; and  (c) may have a distribution system that has up to 14 service connections or up to 4 delivery sites; |
