# Supplementary material for: A Systematic Review of Waterborne Disease Outbreaks Associated with Small Non-Community Drinking Water Systems in Canada and the United States
Source: PLoS One. 2015 Oct 29;10(10):e0141646. doi: 10.1371/journal.pone.0141646 (PMC4625960; doi:10.1371/journal.pone.0141646)
Supplement: S2 Table — (DOC) [file pone.0141646.s003.doc]

| **Question** | **Options** | **Definitions/additional notes** |
| --- | --- | --- |
| RefID |  |  |
| 1. Does the abstract investigate or discuss waterborne outbreak(s) in SDWS in Canada or USA? | 1) Yes, within the context of primary research OR surveillance summaries.  2) Unsure, the type of water system or type of data available cannot be determined from the abstract alone  3) No, none of the above.  *Reviewer Decision:*  *If the reviewer selects option 1) or 2) the article will advance to data extraction.*  *If option 3) is selected, the abstract will be EXCLUDED.* | ***Waterborne outbreak***  is defined as cases of a suspected or  confirmed acute illness related to exposure to biological agents from drinking water that involve two or more individuals. (Single case reports will be excluded).  ***SDWS*** *is defined as* a privately or publically owned system that provides drinking water to the visiting public  The system must provide water for the public (no private residences unless the water system serves multiple residences)  May be classified as a non-community water system (A noncommunity water system serves an institution, industry, camp, park, hotel, or business and can be nontransient or transient. Nontransient systems serve >25 of the same persons for >6 months of the year but not year-round (e.g., factories and schools), whereas transient systems provide water to places in which persons do not remain for long periods (e.g., restaurants, highway rest stations, and parks).  May be classified as a semi-public system (privately owned system providing drinking water to the visiting public)  Is *NOT* a community water system (serving year-round residents of a community, subdivision, or mobile home park) |

**S2 Table. Relevance screening used to identify reported outbreaks in small drinking water systems in Canada and the United States (1970-2014).**
