# Supplementary material for: A Systematic Review of Waterborne Disease Outbreaks Associated with Small Non-Community Drinking Water Systems in Canada and the United States
Source: PLoS One. 2015 Oct 29;10(10):e0141646. doi: 10.1371/journal.pone.0141646 (PMC4625960; doi:10.1371/journal.pone.0141646)
Supplement: S3 Table — (DOC) [file pone.0141646.s004.doc]

**S3 Table. Form used to extract the data from articles reporting outbreaks in small drinking water systems in Canada and the United States (1970-2014).**

Article-level data characterisation and utility form (applicable to all relevance-confirmed articles)

| **Question** | **Options** | **Comments** |
| --- | --- | --- |
| RefID |  |  |
| 1) Is this a surveillance summary, research article or case report on waterborne outbreaks in SDWS in Canada or USA published in English, French or Spanish? | 1) Yes, primary research article/outbreak report/surveillance summary  2) None of the above, specify reason for exclusion   - Not relevant:_______ - Language: _________ - Other:____________   *If 2)“none of the above” is selected, article will be EXCLUDED. Please specify the reason for exclusion and submit the form without completing the remaining questions.* |  |
| 2) Publication Year (YYYY) | __________ |  |
| 3) What is the study design?  *(Check all that apply)* | - Observational study   - Cross-sectional   - Cohort   - Case-control   - Prevalence survey   - Case or case-series    Routine monitoring or surveillance data collection   Outbreak report   Other, please specify:_____________________   Not applicable, this is not primary research | **Observational study**: Assignment of subjects into a treated group versus a control group is outside the control of the investigator.  **Cross-sectional:** Observation of all of a population, or a representative subset, at a defined time  **Cohort study**: is a study in which individuals with differing exposures to a suspected factor are observed over a period of time for occurrence of an outcome  **Case-control study**: compares exposure in patients who have a condition (the 'cases') with subjects who do not have the condition, but are otherwise similar (the 'controls').  **Case or case-series:** a descriptive study of a single individual (case report) or small group (case series).    **Routine monitoring or surveillance**  **data collection:** could primarily cover articles that report on an ongoing and systematic data collection, or the analysis and interpretation of data related to our study question. |
| 4) Do the authors present findings of more than one outbreak in this report? | 1) Yes  2) No | Complete the next questions for each applicable outbreak. |
| 5) Year and month the outbreak occurred (mm/yy) |  |  |
| 6) Water treatment | - Disinfection, specify:___________ - Filtration, specify:___________ - None - Unknown |  |
| 7) Contributing factors leading to the outbreak: | - Weather event - Source contamination (human) - Source contamination (non-human) - Distribution system - Lack of treatment - Treatment failure - Other:________________ - Unknown | Multiple choices permitted. |
| 8) Water source: | - Well - Spring - Surface water, specify:__________ - Other (i.e. cistern or reservoir), specify:________ | Surface water examples include lake, river, pond or reservoir |
| 9) Location of outbreak | - Canada (specify province):_____________ - USA (specify state):______________ |  |
| 10) Premise associated with the outbreak | - School - Motel/hotel - Lodge - Camp - Campground - Park - Restaurant - Trailer park - Resort - Other________________ |  |
| 11) Is the water system seasonal? | - Yes, specify time in use:_________ - No - Unknown | Seasonal water systems operate for less than 12 months per year |
| 12) What definitions are explicitly provided by the authors to describe the water system size in this article? *(Check all that apply)* | 1) Provided by authors:   - Term used to describe the water system size, specify: _______________ - # of connections in the water system, specify:________ - Flow rate of the system, specify:_______________ - # of people served by the water system, specify: __________   2) No definitions provided | **Provided by authors:**   - Please type or copy and paste sentences used to describe these terms. |
| 13) Microorganism involved: | - Giardia - Norwalk/Norovirus - Salmonella - Campylobacter - E.coli - Shigella - Cryptosporidium - Other:______________ - Unknown |  |
| 14) Size of the outbreak: | Confirmed:_____________  Presumptive cases:______  Deaths:__________ |  |
| 1. How was the outbreak determined to be associated with the SDWS? | - Questionnaires/ interviews - Stool samples - Water samples - Other:___________________ | Multiple choices permitted |
| 1. Other |  | Add any comments you think might be useful |
